# Lexical and Buffer Effects in Reading and in Writing Noun-Noun Compound Nouns

**DOI:** 10.3233/BEN-2012-119007

**Published:** 2012-03-19

**Authors:** Sara Mondini, Giorgio Arcara, Carlo Semenza

**Affiliations:** ^1^Department of General PsychologyUniversity of PaduaPaduaItaly; ^2^Casa di Cura Figlie di San CamilloCremonaItaly; ^3^Department of NeuroscienceUniversity of Padua and I.R.C.C.S. Ospedale S. CamilloLido di VeneziaItaly

**Keywords:** Compound nouns, phonological dyslexia, phonological dysgraphia, graphemic buffer

## Abstract

Reading and writing Noun-Noun compound nouns was investigated in two Italian aphasic patients: one with phonological dyslexia and the other with phonological dysgraphia. The patients were required to read, write and repeat a list of Noun-Noun compounds and length-matched non-compound nouns. The dyslexic patient RF read compounds better than non-compounds, and his repetition was flawless for both categories. The dysgraphic patient DA wrote non-compounds better than compounds because of a deficit in keeping separate entries at the lemma level. Differential performance when processing compounds and non-compounds is the result of a deficit in different components within the mental lexicon architecture.

## 1. Introduction

How does the brain combine words to obtain other words? The study of word compounding, i.e. the linguistic mechanism that allows word formation by combining two or more independent words, is an emerging field in neuropsychology. Most data comes from oral naming of pictures in aphasia. Only a handful of studies report on the production of compounds in reading aloud or writing on dictation [1]. Thus, very little is known about compound processing in such tasks.

Extant studies report patients affected by phonological dyslexia/dysgraphia. These patients produce omissions, insertions or substitutions of affixes in reading and writing [2–4]. This deficit is interpreted as over-reliance on the lexical route for reading because of damage to the sub-lexical route for reading e.g. phoneme-to-grapheme conversion. The question how complex words are processed via the lexical route raises two possibilities. The first possibility [5] is that morphologically complex words are de-composed into morphemes before lexical access; the alternative full-listing hypothesis [6] assumes that lexical entries are listed in whole-word form. Hybrid models have been proposed where both types of lexical representation, whole-word and de-composed forms are activated in parallel: distributional properties may then determine the process that wins the race [7]. Insofar as omission and substitution errors on single components characterize reading and writing of compounds, phonological dyslexic/dysgraphic patients have provided overwhelming evidence in favor of decomposition. However little is still known about how the processes of decomposition and whole word access interact with post-lexical processes.

A review of the cognitive neuropsychological literature on compound word processing reveals several facts. Firstly, when patients cannot retrieve a name, patients with all major types of aphasia tend to replace compounds with other compounds and simple words with other simple words [8–11]. This compound effect [12] is found in all reports and is also observed on picture naming tasks where nothing in the picture suggests it must be named with a compound. Likewise, patients with aphasia also substitute Noun-Noun compounds with other Noun-Noun compounds, Verb-Noun compounds with other Verb-Noun compounds and they respect such word building in the production of com-

ISSN 0953-4180/12/$27.50 © 2012 – IOS Press and the authors. All rights reserved pound paraphasias. These findings suggest that knowledge of the compound status of a phonological form, of the compound structure with respect to the position of its components, and of word building rules is independent from the knowledge of the phonological form of the compound. Secondly, most errors in aphasia are omissions of one component, substitutions of one or both components or misordering of components. In particular, Italian patients with a verb-relative-to-noun deficit omit or replace the verb component of Italian Verb-Noun compounds that are nouns [13]. This finding is evidence in favor of de-composition in processing: the verb component, in fact, would not be sensitive to the verb deficit if it were not decomposed from the noun component during processing. Thus, on the whole, neuropsychological findings favor theories assuming that complex words like compounds are stored in a de-composed form in the mental lexicon. Finally, no position effect has been found in substitution errors in patients with a naming deficit exclusively impairing compounds and not simple words [14,15]. This is thought to be evidence of essentially simultaneous activation of compound components in retrieval.

One issue in the processing of compounds is that the production of compounds does not depend only on mental representations or processes carried out within the mental lexicon, but also on peripheral events that can modulate central lexical mechanisms that affect the speaker's performance. The role of *buffers* in reading and writing [16], and in particular the common graphemic buffer for reading and spelling have been the subject of several reports [17–19]. Within a cognitive architecture, the *buffer* component is conceived as a working memory system that contains the representation that specifies abstract letter identities and the sequence in which graphemes appear in a word. This system has limited capacity and holds information until further processes come into play.

The buffer component plays a role in reading and writing and because of its limited capacity it is sensitive to the length of a verbal stimulus. Thus, a word length effect on reading or writing is taken as an indication of a disorder in the phonological or the orthographic buffer, respectively [20]. According to Ward's model [16] there could be separate phonological input and output buffers because the input phonemic code, i.e. the word to be spelled is different from the output phonemic code, i.e. the letter names. The model contains a single orthographic lexicon for both reading and spelling and a common orthographic buffer.

A landmark study by Caramazza, Miceli, Villa and Romani [20] proposed a full set of criteria to identify the locus of a deficit in the graphemic output buffer: (a) the buffer has a limited space capacity and errors should be quantitatively and qualitatively identical in all types of tasks, irrespective of the input or output modality, as the graphemic buffer is involved in each of these tasks; (b) errors should mostly consist of graphemic deviations from the target (i.e., substitutions, deletions, additions or transposition of letters); (c) errors should appear in both familiar words and in non-words. Thus, presence of errors should not be affected by the lexical category of stimulus words or by morphological and semantic features. However, these criteria are not always met in case reports in the literature, where further contributions were added. Morphological features have an effect on writing by individuals with graphemic buffer impairment. For example, a case [21] of a French-speaking woman with acquired dysgraphia, whose deficit could be located at the level of the graphemic buffer, showed spelling errors more frequently with irregular than regular words, although the qualitative type of errors was the same in both categories. The authors [21] discussed these findings in terms of a post-lexical requirement for irregular spelling. Representations of irregular words would require attentional resources within the graphemic buffer level for focusing attention on spelling irregularities. This can cause a detriment to the surrounding graphemic constituents (on this point see [19,22]).

Of relevance to the present reports, the graphemic buffer also plays a role in the input part of reading [17–19], in which graphemic representations are input for word recognition that are applied in parallel over the whole graphemic string. Moreover, if damage to the buffer were to interact differently with those processes that involve serial processing (spelling) from those that involve parallel processing (reading) of the graphemes held in the buffer, one would expect damage to this mechanism to have different consequences for reading and spelling [18].

The interaction between linguistic performance and short-term memory capacity is clear when considering patients with general cognitive decline (e.g., patients with senile dementia of Alzheimer type, SDAT). A recent study [11] compared performance of SDAT patients with a group of aphasic patients on a picture naming task to compare performance of the two groups. Patients with SDAT produced omissions and substitutions more frequently on the second component of compound words when they produced compound paraphasias, whereas aphasic patients produced most errors on the first constituent. This study [11] was the first to examine SDAT patients in compound noun naming, highlighted the emergence of processes intervening during compound retrieval. In particular, the detriment of the second component seemed to reflect a position effect across types of compounds. According to the authors [11], the second portion may be more sensitive to processing overload and thus pose specific problems to SDAT patients.

Badecker, Hillis and Caramazza [23] studied deficits to the production of compounds in the graphemic buffer. Their patient (DH) wrote compounds better than length-matched mono-morphemic words. The authors suggested that compounds had an advantage at the level of the graphemic output buffer since compounds can be stored in de-composed form, i.e. as smaller units that are not greatly affected by the weakening buffer capacity. Furthermore, whereas in the mono-morphemic words the spelling errors were distributed on the final part of the nouns, the errors on compounds fell in the final positions of both the first and the second component. This finding suggests that compounds pass from the orthographic lexicon to the graphemic output buffer in morpheme-sized units. This might lead to the conclusion that composition happens in the buffer not in the lexicon. However, the process must be driven by information about the whole word in the lexicon. That said, a theory about functioning of the buffer is yet to be specified. Here we report two Italian-speaking patients, one dyslexic and one dysgraphic, who contribute to the understanding of compound processing by highlighting the interaction between compositional processes, whole-word access and the capacity of the graphemic buffer.

## 2. Method

A list of Noun-Noun compound and non-compound words and non-words was prepared and administered for reading aloud, writing to dictation and repetition. The stimuli comprised 24 Noun-Noun compounds (NN: 12 left-headed, Nn, e.g., *pescespada*, 'swordfish'; 12 right-headed, nN, e.g., *videogioco*, 'videogame') and 24 non-compounds (NC), mono-morphemic nouns that contained an embedded word homograph and homophone to a word, either in the initial position (NC1, e.g., *pellegrino*, 'pilgrim', where *pelle*, 'skin' is a real word while *grino* is a non-word) or in the final position (NC2, e.g., *pavimento,* 'floor', where *pavi* is a non-word while *mento*, 'chin' is a word). The embedded word was not related in meaning to the whole word. Twelve four-syllable NC with a phonological structure resembling real compounds (e.g., *damigiana*, 'demijohn') were used as control fillers. Non-words were created by exchanging the positions of either morpheme of the NN (e.g., $pesce_1 spada_2$, [lit.] 'fish sword', became the non-word $spada_2 pesce_1$), or the two parts of NC (e.g., the $pelle_1 grino_2$, 'pilgrim', became the non-word $grino_2 pelle_1$). All stimuli are reported in Appendix. Values for the age of acquisition, familiarity, frequency, imageability and length (number of letters) for all stimuli were collected. Word frequency was calculated via a written corpus of over 23 million words from Italian web sites. Values were log-transformed before entering the analysis. Age of acquisition, familiarity and imageability were collected through the administration of three questionnaires to three different groups. Participants of each group ($n = 30$) were asked to rate the words on a 5-point Likert scale (see also [24]).

Four categories of words were considered: left-headed NN, right-headed NN, NC with an embedded word in the left position and NC with an embedded word in the right position. Across items there was no difference in familiarity frequency, imageability or length. However, NN items were acquired significantly later than NC ($p < 0.001$). Moreover, within compounds left-headed NN items were acquired earlier than right-headed NN ($p < 0.001$). Table 1 shows the mean values for each variable across categories.

## 3. Results

### 3.1. Case 1: A patient with phonological dyslexia

RF was a 31 year old, right-handed, Italian-speaking man, with eight years of education. In 2006 (one year before the study) he underwent surgical treatment for an angioma bleeding into the left fronto-temporo-parietal region. The aphasia examination (Italian version of the AAT [25], Table 2) revealed non-fluent spontaneous speech and spared comprehension. On the ENPA (Esame Neuropsicologico per l'Afasia, *tr.* Neuropsychological examination of aphasia [26]), RF showed spared repetition of both words (100% correct) and non-words (100% correct), whereas oral reading performance with words (11/13, 85% correct) was better than with non-words (0/15); he also demonstrated a grammatical class effect whereby nouns were read better than adjectives (15/20, 75% correct and 6/20, 30%

Table 1
Psycholinguistic characteristics of experimental items

| Type | N = | Example | Length | Familiarity | Log. Freq | AOA | Imag |
|---|---|---|---|---|---|---|---|
| Nn | 12 | *Capobanda,* 'band leader' | 9.92 (0.79) | 5.11 (0.77) | 2.20 (0.31) | 4.64 (0.97) | 5.15 (1.00) |
| nN | 12 | *Videogioco* 'videogame' | 10.75 (1.71) | 5.01 (0.90) | 2.41 (0.40) | 5.09 (0.98) | 5.06 (1.00) |
| NC1 | 12 | *Coccodrillo* 'crocodile' | 9.75 (1.66) | 5.67 (1.22) | 2.08 (0.57) | 3.74 (0.69) | 5.20 (0.88) |
| NC2 | 12 | *Accidente* 'accident' | 9.25 (0.75) | 5.84 (0.99) | 1.97 (0.68) | 3.91 (0.66) | 5.09 (0.94) |

Nn: left-handed compounds; nN: right-handed compounds; NC1 non-compounds with a word embedded in the left part of the whole word; NC2: non-compounds with a word embedded in the right part of the whole word; Length: Number of Letters. Log. Freq: Log-transformed Frequency; AOA: Age of Acquisition; Imag: Imageability.

Table 2
Results from Achener Aphasia Test for patient RF

| | Raw score | PR | T | Deficit |
|---|---|---|---|---|
| Token | 7 | 92 | 64 | Slight/minimal |
| Repetition | 145 | 94 | 66 | Slight/minimal |
| Written language | 86 | 96 | 67 | Slight/minimal |
| Naming | 115 | 100 | 80 | Minimal |
| Comprehension | 120 | 100 | 80 | Minimal |

PR: Percentile Rank. T: T-score.

correct), verbs (8/20, 40% correct) and function words (7/20, 35% correct). RF read simple words i.e. in the citation form better than inflected words (16/20 correct 80% *vs.* 9/20, 45%). The reading of a list of thirty words varying in length (range 4–12 letters) showed that as the number of letters increased, the number of correct responses decreased (length, $\beta = -0.947$, Wald $Z = -3.05$, $p < 0.005$). Unfortunately, RF's writing proved hard to investigate. The patient, who used the non-dominant left hand and had additional writing apraxia problems, could only produce a few ill-formed scribbles that were not easily interpretable. For this reason this investigation concerned only repetition and reading.

RF was required to repeat and to read aloud the experimental stimuli i.e. compounds and NC and non-words described in the Method section. His performance was tape-recorded and later analyzed in order to assess reading strategies for each item type. RF's repetition was flawless for all items: compounds, NC and all non-words. RF read aloud correctly 51/60 (85%) words and 19/60 (46.3%) non-words $\chi^2(1) = 35.1$, $p < 0.001$ thus conforming to a pattern of phonological dyslexia. Errors were mostly substitutions or omissions of letters, especially in the second half of a stimulus. Within words, RF read all compounds flawlessly (24/24, 100% correct), but was less effective reading NC (17/24, 70.8%), $\chi^2(1) = 8.19$; $p < 0.01$. With matched-for-length NC, and non-words, reading was effortful and slow. Table 3 summarizes his errors.

In the case of non-words, RF read correctly 13/24 (54.16%) inverted compounds and only 5/24 (20.8%) non-words derived from NC $\chi^2(1) = 5.69$, $p = 0.02$. Furthermore, whereas in oral reading inverted NC, the patient read slowly and often in a "letter-by-letter" fashion (in 13/24 cases), this strategy appeared less frequently with inverted compounds (in 4/24 cases), $\chi^2(1) = 4.75$, $p = 0.02$. In inverted compounds, RF made a peculiar kind of error in five cases out of nine errors: hyper-lexicalization of the written stimulus, i.e. he reversed the order of constituents and uttered the correct compound e.g., the inverted compound $maglia_2calza_1$ was read reversing the constituents and saying the correct compound $calza_1maglia_2$ 'tights'; and $banda_2capo_1$ was read correctly as $capo_1$ $banda_2$, 'band leader'. Thus, RF segmented non-words and re-composed them as real words.

RF read compounds better than length-matched NC; furthermore within non-words, he read inverted compounds better than inverted NC. The fact that he could repeat easily and without errors both compounds and NC, makes the possibility of a deficit at the phonological output buffer unlikely. Instead, the locus of impairment could be reasonably placed at the level of the graphemic buffer, in line with the proposal that such a processing component plays a role in reading [19,20]. According to this account, when reading aloud compounds, RF could retain separate abstract morphemes in the buffer, then access the orthographic lexicon for both the lexical units. This interpretation is analogous to the graphemic output buffer deficit reported by Badecker et al. [23]. In contrast, matched-for-length NC, which cannot be de-composed into two real words, put an overwhelming load on a defective buffer i.e. when separated, the non-lexical strings cannot retrieve lexical entries. Moreover, the fact that compound constituents

Table 3
Reading errors for RF

| Category | N | Example | Errors | | | | |
|---|---|---|---|---|---|---|---|
| | | | L by L | − + | + − | − − | TOT |
| Nn | 12 | *pescespada,* 'swordfish' | | | | | 0 |
| nN | 12 | *videogioco,* 'wordgame' | | | | | 0 |
| NC1 | 12 | *pellegrino,* 'pilgrim' | 3 | 1 | | | 4 |
| NC2 | 12 | *pavimento,* 'floor' | 2 | 1 | | | 3 |
| *TOT* | *48* | | *5* | *2* | | | *7* |

Nn: left-headed compounds; nN: right-headed compounds; NC1: non-compounds containing an embedded word in the initial position; NC2: non-compounds containing an embedded word in the final position.
Errors: (L by L) "letter-by-letter" strategy when reading a whole word; (− +) deletion or substitution of first constituent; (+ −) deletion or substitution of second constituent; (− −) deletion or substitution of both constituents of the word.

have a higher lexical frequency than whole compounds facilitates recognition of singular components.

Hyper-lexicalizations of inverted compounds are an interesting feature of this case. These errors could be explained in two different ways. First, to reduce the overwhelming load, RF may divide inverted compounds in two parts before they enter the buffer. Inverted NCs thus access the lexicon in a decomposed form. Subsequently, RF may access the phonological form at the *word form* level where the correct order of constituents is assigned [3,27]. Alternatively (according to the *dual route model*) both constituent forms and the whole-word are available in the lexicon [7,28]. Thus, after recognizing each constituent, RF can occasionally retrieve the only whole-word compound available in the lexicon, which will be in the correct order.

### 3.2. Case 2: A patient with phonological dysgraphia

DA is 82 years old, an Italian-speaking woman, right-handed, with thirteen years of education. She suffered a cerebro-vascular accident resulting in an ischemic lesion in the left corona radiata. The aphasia examination (Italian version of the AAT [25] Table 3) revealed fluent spontaneous speech with anomia i.e. difficulty in retrieving the whole phonological form of items mainly in the compound naming section of the battery and spared comprehension of short sentences. Reading and repetition abilities were well preserved except for long phrases. Writing was impaired: showing a clear pattern of phonological dysgraphia, with word over non-word superiority, emerged from the assessment (16/20, 80% and 4/20, 20%, $\chi^2(1) = 14.4$, $p < 0.001$). There was an effect of grammatical class on writing: nouns written better than adjectives (14/20, 75% correct and 7/20, 30% correct, $\chi^2(1) = 4.91, p = 0.03$), verbs (7/20, 35% correct $\chi^2(1) = 4.91$, $p = 0.03$) and function words (7/20, 35% correct, $\chi^2(1) = 7.15, p = 0.007$). Furthermore, DA wrote simple words in citation form better than inflected words (16/20 correct 80% *vs.* 6/20, 30%, $\chi^2(1) = 10.10, p = 0.001$).

Table 4
Results from Achener Aphasia Test for patient DC

| | Raw score | PR | T | Deficit |
|---|---|---|---|---|
| Token | 23 | 58 | 52 | Medium/slight |
| Repetition | 146 | 95 | 67 | Slight/minimal |
| Written language | 82 | 92 | 64 | Slight/minimal |
| Naming | 96 | 82 | 59 | Slight/minimal |
| Comprehension | 105 | 84 | 60 | Slight/minimal |

PR: Percentile Rank. T: T-score.

DA was administered the list of compounds, NC, fillers and non-words in a writing on dictation task. The examiner read each item aloud in a neutral tone, i.e. without underlining the presence of two separate words in the case of compound nouns. Furthermore, DA was administered a list of 48 pairs of words with the conjunction $e$ (and), interposed. Twenty-four pairs were made up of two morphemes of each compound stimuli e.g., for the compound *capobanda,* 'band leader', the corresponding pair was *capo e banda,* 'leader and band', and 24 pairs of two-syllable words matched for length and frequency with the compound constituents e.g., *canto e filtro*, 'song and filter'.

DA wrote 28/58 (48.3%) words and 11/58 (18.9%) non-words $\chi^2(1) = 11.16$, $p < 0.01$, confirming the classical pattern of phonological dysgraphia (two NC items were deleted from the list of 60 NC described in the Method section). Within words, DA wrote NC (14/22, 64%) significantly better than compounds

Table 5
Reading errors for DA

| Category | N | example | Errors | | | | |
|---|---|---|---|---|---|---|---|
| | | | L by L | − + | + − | − − | TOT |
| Nn | 12 | *pescespada,* 'swordfish' | 1 | | 5 | | 6 |
| nN | 12 | *videogioco,* 'wordgame' | 2 | | 9 | | 11 |
| NC1 | 11 | *pellegrino,* 'pilgrim' | | 2 | 3 | | 5 |
| NC2 | 11 | *pavimento,* 'floor' | 1 | 1 | | 1 | 3 |
| *TOT* | *46* | | *4* | *3* | *17* | *1* | *25* |

Nn: left-headed compounds; nN: right-headed compounds; NC1: non-compounds containing an embedded word in the initial position; NC2: non-compounds containing an embedded word in the final position).
Errors: (L by L) "letter-by-letter" strategy when reading a whole word; (− +) deletion or substitution of first constituent; (+ −) deletion or substitution of second constituent; (− −) deletion or substitution of both constituents of the word.)

(7/24, 29.1%; $\chi^2(1) = 5.5$, $p = 0.02$), in which errors e.g., deletions, substitutions always fell on the second constituent regardless of headedness. Table 5 shows DA's errors when writing words. In the second condition, when compound constituents were dictated as two separate words mixed with other pairs of words DA made no errors and wrote flawlessly all pairs of words.

NC writing was better than compound writing. Impaired processing of compounds and preserved processing of NC points to a deficit in the retrieval of compounds in the orthographic lexicon. Given the phonological dysgraphia, DA must use the lexical routine. Her deficit can be explained by positing a difficulty in keeping trace of both constituents of the compound.

Another aphasic patient with impairment in the retrieval of compound nouns in a picture naming task has been reported [14]. The authors explained her impairment within the frame of a dual-stage model [3,27] according to which the first step is the selection of the lexical item and the assignment of semantic and syntactic features. Subsequently, phonological features are retrieved. Compound nouns are generated by combining two lexical entries at the first stage. The authors suggest that the deficit in their patient arose when two different constituents were defined by a single lexical entry (one compound noun). This impairment is similar to the deficit shown by DA in writing and the same interpretation could apply. What remains to be explained is where composition process takes place. DA could not compose compound constituents and only occasionally find the whole-word compound form within the lexicon. Impairment to the compositional process is confirmed by the observation that she could write both constituents of a compound when they were dictated as two separate words with an interposed conjunction. In this condition, in fact, DA did not have to do any morphological composition. Separation of the constituents increased the total length of the stimulus (*capo e banda* in place of *capobanda*), but, on the other hand, spread the charge over smaller sub-units that could be held in the buffer.

## 4. General discussion

The performance of patients with acquired dyslexia and dysgraphia in reading/writing NN compound nouns highlights interactions between lexical and peripheral processes required for processing compound words. RF, the patient with phonological dyslexia, read compound nouns better than non-compounds. This performance is surprising for a phonological dyslexic, who usually fails to read morphologically complex words [3]. However, RF does not have any problem with composition in reading as in previously described patients. Compound word representations in the lexicon are strong, despite phonological dyslexia. RF's performance can be explained in light of the limited capacity of the deficient graphemic buffer. Another patient with phonological dysgraphia (DH) [23] writes compound nouns significantly better than monomorphemic nouns. This performance was interpreted to result from a deficit at the graphemic output buffer as compound nouns could be divided in morpheme-sized units and it was easier to hold them in a defective buffer. RF seems to match in reading, DH's performance in writing. Phonological dyslexia caused more difficulties in reading morphologically complex words, but a defective graphemic buffer took advantage of long compound nouns decomposed into small units (compound constituents). Our case provides converging evidence that morphologically complex words access the output buffer in a decomposed form.

In contrast, the impaired writing of compounds with respect to non-compounds found in DA points to another possible locus of functional impairment. The analysis of her performance allows us to hypothesize a deficit in compositional processes, in particular keeping different representations in one lexical entry. Delazer and Semenza [14] reported similar impairments to picture naming (patient MB). We contend that whereas the impairment for the dyslexic patient RF was located at the graphemic buffer, there was a lexical impairment for DA specifically a deficit to morphologically complex words affecting compounding. Indeed, if compounds were de-composed in two single words, DA wrote these items flawlessly.

The patients reported here differ in their processing of compounds: selectively spared in one case (RF) and selectively affected in the other (DA). The comparison (including reference to other patients DH and MB) between the two cases is useful because it illuminates aspects of compounding that arise and interact at different levels of retrieval. The first process is driven by lexical information about a whole word wherein constituents are chosen and arranged in the correct order. However at this stage, compound words are apparently not yet assembled, and as RF and DH show, they are delivered as independent units. The next step – assembly – occurs in the buffer. If the buffer is defective, as in RF (and DH), production of compounds is better than monomorphemic words. However if constituent selection fails, monomorphemic words will be retrieved in favour of compounds, as observed in DA (and MB). Cases of acquired dyslexia and dysgraphia clearly have the potential to stimulate future research and discussion in the field of compound word processing.

## Appendix. Experimental stimuli

| Category | Stimulus | Translation | Translation of stimulus constituents |
|---|---|---|---|
| Nn | *Arcobaleno* | Rain-bow | *arco* (bow); *baleno* (lightning) |
| Nn | *Bancoposta* | [lit.] Counter post (the post office counter) | *banco* (counter); *posta* (post) |
| Nn | *Calzamaglia* | Tights | *calza* (sock); *maglia* (knitting) |
| Nn | *Camposcuola* | School camp | *campo* (camp); *scuola* (school) |
| Nn | *Capobanda* | Band leader | *capo* (leader); *banda* (band) |
| Nn | *Ceralacca* | Sealing wax | *cera* (wax); *lacca* (lake) |
| Nn | *Finecorsa* | Terminal station | *fine* (end); *corsa* (run) |
| Nn | *Fondovalle* | Valley bottom | *fond* (bottom); *valle* (valley) |
| Nn | *Girocollo* | Round neck | *giro* (round); *collo* (neck) |
| Nn | *Gommapiuma* | Foam rubber | *gomma* (rubber); *piuma* (feather) |
| Nn | *Metroquadro* | Square metre | *metro* (metre); *quadro* (square) |
| Nn | *Pescespada* | Swordfish | *pesce* (fish); *spada* (sword) |
| nN | *Astronave* | Spaceship | *astro* (star); *nave* (ship) |
| nN | *Calciomercato* | [lit.] Soccer market | *calcio* (soccer); *mercato* (market) |
| nN | *Cartamoneta* | Paper money | *carta* (paper); *moneta* (money) |
| nN | *Crocevia* | Crossroads | *croce* (cross); *via* (road) |
| nN | *Docciaschiuma* | Shower gel | *doccia* (shower); *schiuma* (foam) |
| nN | *Fangoterapia* | Mud therapy | *fango* (mud); *terapia* (therapy) |
| nN | *Fotoromanzo* | Picture story | *foto* (photograph); *romanzo* (romance) |
| nN | *Madrepatria* | Motherland | *madre* (mother); *patria* (land) |
| nN | *Mondovisione* | World vision | *mondo* (world); *visione* (vision) |
| nN | *Montepremio* | Jack-pot | *monte* (mountain); *premio* (prize) |
| nN | *Motosega* | Chain saw | *moto* (motor); *sega* (saw) |
| nN | *Radiocronaca* | Running commentary | *radio* (radio); *cronaca* (commentary) |
| nN | *Vetroresina* | Fibre-glass plastic | *vetro* (glass); *resina* (resin) |
| nN | *Videogioco* | Videogame | *video* (video); *gioco* (game) |
| NC1 | *Cavaliere* | Horse-rider | *cava* (mine) |
| NC1 | *Clorofilla* | Chlorophyll | *cloro* (chloro) |
| NC1 | *Coccodrillo* | Crocodile | *cocco* (coconut) |
| *NC1 | *Cremagliera* | Rack | *crema* (cream) |
| NC1 | *Filastrocca* | Rigmarole | *fila* (row) |
| NC1 | *Gelosia* | Jealousy | *gelo* (chill) |
| NC1 | *Maresciallo* | Marshal | *mare* (sea) |
| NC1 | *Melodia* | Melody | *melo* (apple-tree) |
| NC1 | *Pellegrino* | Pilgrim | *pelle* (skin) |
| NC1 | *Peperone* | Pepper | *pepe* (pepper) |
| NC1 | *Polpastrello* | Pulp | *polpa* (pulp) |
| NC1 | *Salamandra* | Salamander | *sala* (hall) |
| NC1 | *Serratura* | Lock | *serra* (greenhouse) |
| NC1 | *Temperatura* | Temperature | *tempera* (distemper) |
| NC2 | *Accidente* | Accident | *dente* (tooth) |
| *NC2 | *Catafalco* | Catafalque | *falco* (hawk) |
| NC2 | *Dirigente* | Manager / director | *gente* (people) |
| NC2 | *Fazzoletto* | Handkerchief | *letto* (bed) |
| NC2 | *Logaritmo* | Logarithm | *ritmo* (rythm ) |
| NC2 | *Marzapane* | Marzipan | *pane* (bread) |
| NC2 | *Pavimento* | Floor | *mento* (chin) |
| NC2 | *Scarafaggio* | Cock-roach | *faggio* (beech tree) |
| NC2 | *Schiamazzo* | Din | *mazzo* (bunch) |
| NC2 | *Tartaruga* | Tortoise | *ruga* (wrinkle) |
| NC2 | *Varicella* | Chicken pox | *cella* (cell) |
| NC2 | *Vegetale* | Vegetable | *tale* (someone) |

Nn: left headed compounds; nN: right headed compounds; NC1: non-compounds with a real word embedded on the left side of the whole word; NC2: non-compounds with a real word embedded on the right side of the whole word (letter strings corresponding to real Italian words are translated).
*Items not used in the writing on dictation task administered to DA.
